# A152 NATURAL HISTORY OF SMALL BOWEL STRICTURES IN CROHN’S DISEASE

**DOI:** 10.1093/jcag/gwab049.151

**Published:** 2022-02-21

**Authors:** D C Vu, G S Brar, K Dadgar, J McCurdy

**Affiliations:** 1 Gastroenterology, The Ottawa Hospital, Ottawa, ON, Canada; 2 University of Toronto, Toronto, ON, Canada; 3 University of Ottawa, Ottawa, ON, Canada

## Abstract

**Background:**

Crohn’s disease (CD) is a progressive inflammatory disease that often results in intestinal complications such as small bowel (SB) strictures. SB strictures are frequently associated with substantial morbidity and may require surgery. The natural history of SB strictures in the era of biologic treatments has not been well characterized.

**Aims:**

To determine the proportion of patients with SB strictures who develop complicated stricturing disease and to identify clinical factors associated with this outcome.

**Methods:**

We performed a retrospective observational study between January 1, 2009, and May 31, 2019. Adults (>17 years) with CD who underwent an abdominal CT scan or MRI were identified from our institutional data warehouse using the ICD-10 code K50* and local imaging codes. Reports were reviewed to determine the imaging protocol and the presence of SB strictures. We included CT or MR enterography studies that reported SB strictures and excluded encounters with incomplete records, diverting ostomies, ileal J-pouches, and patients evaluated for pre-surgical planning. Each imaging study was included as a separate encounter in our analysis. Our primary endpoint was the development of complicated stricturing disease defined as stricture-related hospitalization or surgery. Time to event was estimated using Kaplan–Meier analysis and associated factors were assessed by multivariable Cox proportional hazard models adjusted for age, sex, exposure to biologics and corticosteroids.

**Results:**

A total of 6583 unique imaging studies were identified: 926 (14%) studies reported SB strictures without penetrating complications, and 568 (9%) studies reported penetrating complications. A total of 503 (8%) studies, performed on 330 patients, met our inclusion criteria: mean age 42 (SD, 15.1) years and 166 (50%) males. Overall, 144 (44%) patients developed complicated stricturing disease: 106 (32%) patients required surgery and 132 (40%) patients were hospitalized for stricture related complications. Of the patients who underwent surgery, the mean time to surgery was 13 months (SD, 18.3) and among patients who required hospitalization, mean time to hospitalization was 15 months (SD, 17.8) (Figure 1). On multivariable analysis, exposure to corticosteroids (aHR 2.11; 95% CI, 1.54–2.90; p<0.001) but not biologics (aHR 1.1; 95% CI, 0.84–1.43; p<0.48) at the time of the imaging study was independently associated with the development of complicated stricturing disease.

**Conclusions:**

In our single center study, complicated stricturing disease occurred in 44% of patients with CD who had a SB stricture and was associated with corticosteroids but not biologics.These findings, along with additional clinical and radiologic factors may help in the development of clinical support tools to identify patients at highest risk of developing complicated stricturing disease.

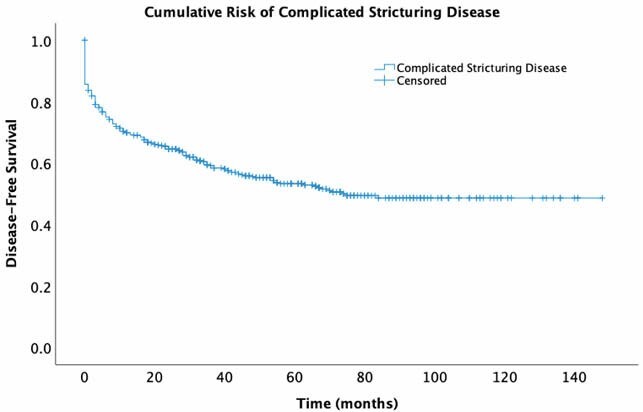

Figure 1. Kaplan-Meier estimates of the time to complicated stricturing disease (surgery or hospitalization) after an imaging encounter documenting SB stricture(s).

**Funding Agencies:**

None

